# Prevalence of Self-Reported Diabetes and Exposure to Organochlorine Pesticides among Mexican Americans: Hispanic Health and Nutrition Examination Survey, 1982–1984

**DOI:** 10.1289/ehp.10258

**Published:** 2007-10-02

**Authors:** Shanna Cox, Amanda Sue Niskar, K.M. Venkat Narayan, Michele Marcus

**Affiliations:** 1 Department of Epidemiology and; 2 Department of Environmental and Occupational Health, Rollins School of Public Health, Emory University, Atlanta, Georgia, USA; 3 Environmental Tracking Branch, Division of Environmental Hazards and Health Effects, National Center for Environmental Health, Centers for Disease Control and Prevention, Atlanta, Georgia, USA; 4 Department of Environmental and Occupational Health, School of Public Health, Tel Aviv University, Tel Aviv, Israel; 5 Division of Diabetes Translation, National Center for Chronic Disease and Heath Promotion, Centers for Disease Control and Prevention, Atlanta, Georgia, USA; 6 Hubert Department of Global Health, Rollins School of Public Health, Emory University, Atlanta, Georgia, USA

**Keywords:** diabetes, health effects, HHANES, Hispanic, organochlorines, pesticides

## Abstract

**Background:**

The prevalence of diabetes is higher among Mexican Americans than among non-Hispanic whites. Higher serum levels of organochlorine pesticides in Mexican Americans have been reported. Few studies have explored the association between pesticide exposure and diabetes.

**Objectives:**

We set out to examine the association between self-reported diabetes and serum concentrations of organochlorine pesticides among Mexican Americans residing in the southwestern United States from 1982 to 1984.

**Methods:**

This study was conducted among a sample of 1,303 Mexican Americans 20–74 years of age from the Hispanic Health and Nutrition Examination Survey. Serum concentrations were available for seven pesticides or pesticide metabolites at quantifiable levels in at least 1% of the study population: *p,p*′-DDT (dichlorodiphenyltrichloroethane), *p,p*′-DDE (dichlorodiphenyldichloro-ethylene), dieldrin, oxychlordane, β-hexachlorocyclohexane, hexachlorobenzene, and *trans*-nonachlor. We used logistic regression to evaluate the association of self-reported diabetes with exposure to organochlorine pesticides, with and without adjustment for total serum lipids. Nonfasting serum glucose values were compared among exposure groups.

**Results:**

Self-reported diabetes was significantly associated with serum levels above the detectable limit for *trans*-nonachlor, oxychlordane, and β-hexachlorocyclohexane and among those with the highest level of exposure to *p,p*′-DDT and *p,p*′-DDE. On adjustment for total serum lipids, the association with *p,p*′-DDT remained significant. Serum glucose levels were elevated among those exposed to *trans*-nonachlor and β-hexachlorocyclohexane.

**Conclusion:**

This study suggests that higher serum levels of certain organochlorine pesticides may be associated with increased prevalence of diabetes. Additional studies with more extensive clinical assessment are needed to confirm this association.

A Hispanic individual in the United States has one chance in two of developing diabetes sometime during his or her lifetime (Narayan et al. 2003). Hispanics represent the fastest-growing minority group in the United States. Among the Hispanic population, two-thirds are of Mexican origin (Ramirez and de la Cruz 2002). The prevalence of diabetes is twice as high for Mexican Americans than for non-Hispanic whites (Haffner 1998).

In the United States, serum levels of organochlorine pesticides and metabolites are highest in Mexican Americans and those who reside in the southwestern regions of the United States [Centers for Disease Control and Prevention (CDC) 2005; Stehr-Green 1989]. The U.S. Environmental Protection Agency (EPA) banned most uses of these compounds during the 1970s and 1980s, and serum levels of organochlorine pesticides in the United States have decreased over time; however, exposure among the general population persists through bioaccumulation of these compounds in the food chain (CDC 2005). DDT (dichlorodiphenyltrichloroethane) continues to be used for malaria vector control in parts of Africa and India.

Evidence of an association between persistent environmental contaminants and diabetes has begun to accumulate. Accidental environmental releases (Pesatori et al. 1998) and occupational exposures related to the manufacture or application of organochlorines has been associated with diabetes morbidity and mortality (Beard et al. 2003; Laws et al. 1967; Morgan et al. 1980; Vena et al. 1998). Limited and inconclusive data have linked higher serum levels of polychlorinated biphenyls (PCBs) with increased risk of type I diabetes (Longnecker et al. 2001). In 1999, the Institute of Medicine (2000) concluded that there was limited, suggestive evidence of an association between exposure to herbicides contaminated with 2,3,7,8-tetrachlorodibenzo-*p*-dioxin (TCDD) and type II diabetes. A study among an elderly Swedish cohort found that diabetics had significantly higher serum levels of persistent organochlorine pollutants than nondiabetic control subjects (Rylander et al. 2005). More recently, cross-sectional studies of a nationwide probability sample representative of the general population of the United States have found a strong association between diabetes and a range of persistent environmental contaminants including dioxins, PCBs, and organochlorine pesticides (Everett et al. 2006; Lee et al. 2006). Serum levels of PCBs measured before the development of adult-onset diabetes among a cohort of Michigan women were significantly associated with diabetes incidence (Vasiliu et al. 2006).

Because exposure to persistent environmental contaminants differs by place and has changed over time, population and period effects are plausible (Porta 2006). The association between diabetes and exposure to persistent organic pollutants was found to be stronger among Mexican Americans (Lee et al. 2006) than among non-Hispanic whites or African Americans. The Hispanic Health and Nutrition Examination Survey (HHANES) is the largest and most comprehensive Hispanic health survey in the United States (Delgado et al. 1990). This cross-sectional study investigates the relationship between individual serum organochlorine pesticide concentrations and the prevalence of self-reported diabetes among the 1980 Mexican-American population residing in the southwestern region of the country.

## Materials and Methods

### Study population

The HHANES was conducted by the CDC National Center for Health Statistics in 1982–1984 (Delgado et al. 1990). The HHANES was a stratified multistage probability sample of three Hispanic subgroups of the population: Mexican Americans residing in Arizona, California, Colorado, New Mexico, and Texas; Puerto Ricans residing in the New York City metropolitan area (including parts of New York, New Jersey, and Connecticut); and Cuban Americans residing in Dade County, Florida (Delgado et al. 1990). The sampling frame included approximately 76% of the 1980 Hispanic origin population in the United States (Delgado et al. 1990). This publicly available de-identified database is unique in its ability to provide comprehensive health information on Hispanic Americans. A description of the HHANES sample design and operational plan has been published [Department of Health and Human Services (DHHS) 1985].

The family unit determined eligibility for inclusion into the survey. Eligible families were those in which at least one member was identified as being Mexican, Mexican American, Chicano, or “Hispano” in the Southwest; Cuban or Cuban American in Dade County, Florida; and Puerto Rican or Boricuan in the New York City area (DHHS 1985). Each household member was then eligible for selection for the interview and examination procedure (sample person), some of whom were non-Hispanic individuals. Informed consent was obtained before data collection from each sample person in accordance with study procedures (DHHS 1985). Seventy-six percent of sampled Mexican Americans completed the both the interview and examination portion of the survey (Delgado et al. 1990).

Data collection began with a household questionnaire, followed by a physical examination (DHHS 1985). Socioeconomic, health, and demographic data were collected by interview. Sample persons were administered a questionnaire by trained bilingual interviewers in either Spanish or English (DHHS 1985).

During the examination component of the survey, a variety of tests and procedures were conducted based on age at interview and other factors (DHHS 1985). Blood samples were obtained by venipuncture for both fasting and nonfasting subjects. Specimens were collected, stored, and subsequently analyzed using uniform laboratory protocols (DHHS 1985). A clinical chemistry profile included serum glucose measured by the hexokinase method (DHHS 1991). Total serum cholesterol and serum triglyceride analyses were performed in zeolite-treated isopropanol extracts using the Liberman-Burchard reaction for cholesterol measurement and a fluorimetric method for triglyceride measurement (DHHS 1991).

The pesticide component of the HHANES is comprised of questionnaire and laboratory data (DHHS 1992). Farm work histories and pesticide exposure data were collected by questionnaire. Serum pesticide concentrations were determined by a collaboration between the U.S. EPA and the CDC (DHHS 1992). Participants 20–74 years of age were assigned either to have a serum pesticide determination or to undergo an oral glucose tolerance test (DHHS 1992). Direct solvent (hexane) extractions followed by electron capture gas chromatography were used to determine blood serum concentrations of chlorinated hydrocarbon pesticides (DHHS 1992). Whole weight serum concentrations of organochlorine pesticides were reported as parts per billion (ppb). This is equivalent to nanograms per gram serum and, when adjusted for lipids, nanograms per gram lipid.

### Statistical analysis

This report is restricted to the Mexican-American portion of the survey to reduce potential confounding by ethnicity because of differential diabetes prevalence by Hispanic ethnic group (Flegal et al. 1991) and regional variation in serum organochlorine pesticide levels (Stehr-Green 1989). Analysis was restricted to sample persons 20–74 years of age who reported being of Mexican or Mexican-American ancestry and whose race (as observed by interviewer) was noted as white. Persons who responded yes to both “Do you have diabetes or sugar diabetes?” and “Did a doctor tell you that you have it?” were defined as having self-reported diabetes (Perez-Stable et al. 1989).

We examined the association between occupations presumed to involve pesticide exposure and prevalence of diabetes. Questions used to identify occupations with the potential for pesticide exposure included “Have you ever worked in a pesticide processing plant?”; “Have you ever worked as a pesticide applicator or sprayer?” and “Have you ever done farm work, either paid or unpaid?” Crude odds ratios (ORs) were calculated.

We analyzed 18 organochlorine pesticides and metabolites using blood serum samples; however, only seven compounds were found above the minimum detectable limit in > 1% of HHANES serum samples and used in this analysis: *p,p*′-DDT, *p,p*′-DDE (dichloro-diphenyldichloroethylene), dieldrin, oxychlordane, β-hexachlorocyclohexane (β-HCH), hexachlorobenzene (HCB), and *trans*-nonachlor. Initially, most organochlorine pesticides were categorized as above or below the minimum detectable limit except for *p,p*′-DDE. More than 99% of the sample had measurable amounts of this chlorinated hydrocarbon. Therefore, we categorized serum concentrations by quartiles reflecting the distribution among persons with self-reported diabetes. Where sufficient sample size permitted, we assessed dose response by grouping organo-chlorine serum concentrations into three categories: below the minimum detectable limit, detection limit up to median concentration among persons with self-reported diabetes, and above the median concentration.

We extracted select demographic variables from the interview questionnaire as potential confounders based on *a priori* knowledge of associations with diabetes and serum organo-chlorine concentrations: age at examination, body mass index (BMI), sex, place of birth (United States vs. foreign born), education, and poverty index (Glynn et al. 2003; James et al. 2002; Laden et al. 1999; Moysich et al. 2002; Schildkraut et al. 1999; Stehr-Green 1989; Wolff et al. 2005). The poverty index is a ratio that compares reported family income to poverty thresholds developed by the U.S. Census Bureau that takes into account family structure; values < 1 indicate living below the poverty level, values ≥ 1 are at or above the poverty level (DHHS 1985).

We used individual logistic regression models to test the association between self-reported diabetes and serum concentrations of each respective organochlorine pesticide. Because of small numbers of cases in some cells, we initially evaluated potential confounders one at a time. In these preliminary analyses, age at examination emerged as the strongest confounder of the OR estimate. Additional variables were added to the model one at a time; those that changed the age adjusted estimate of association appreciably (~ 10%) were investigated further as potential confounders. The goal of this study was to describe the strength of the association between measurable levels of organochlorine pesticides and self-reported diabetes; however, the magnitude of the effect measure may differ by demographic categories. Because of small sample sizes that consequently led to unstable estimates when interaction terms were included in the final model, we explored possible effect modification using stratified analysis.

Organochlorine pesticides are highly lipophilic compounds. In the analysis of these environmental contaminants and human health outcomes, adjustment for total lipids at the time of blood draw has been suggested (Phillips et al. 1989). We computed total lipids with total cholesterol and triglyceride data using the following equation: Total lipids = (2.27 × total cholesterol) + triglyceride + 0.623 (Phillips et al. 1989). Pesticide concentrations above the minimum detectable limit were divided by the corresponding total lipid value, and the association between serum lipid adjusted pesticides and self-reported diabetes was assessed. Serum glucose measurements were transformed by taking the natural logarithm, and mean levels were compared among individuals with different pesticide levels using *t*-tests. Two-sided *p*-values are reported. Mean serum glucose was adjusted for age at examination and BMI.

Analysis was done using SUDAAN version 9.0 (RTI International, Research Triangle Park, NC) to take into account the complex sampling design of HHANES and produce accurate standard errors. The weighted estimates in this report are representative of Mexican Americans residing in the five southwestern states of Arizona, California, Colorado, New Mexico, and Texas.

## Results

The percent of samples above the minimum detectable limit varied by organochlorine pesticide compound, from 4.8% for dieldrin to 99.8% for *p,p*′-DDE (Table 1). Only three compounds were found in > 10% of the sample: *p,p*′-DDT, *p,p*′-DDE, and β-HCH. Many of the organochlorine compounds evaluated were correlated with one another (data not shown). The highest correlations were found among related compounds *trans-*nonachlor/oxychlordane and *p,p*′-DDT/*p,p*′-DDE (Spearman correlation coefficients = 0.51 and 0.53 respectively, *p*-value < 0.0001).

Among the 1980 Mexican-American population in the Southwest 20–74 years of age, 5.5% self-reported diabetes. The association between self-reported diabetes and BMI, age at examination, level of education obtained, alcohol consumption, and poverty index was statistically significant (Table 2). Diabetes was most prevalent among persons categorized as obese (12.1%), persons > 55 years of age at the time of examination (25.6%), and former drinkers (16.4%).

Participants who reported ever working in a pesticide processing plant [crude OR = 3.5; 95% confidence interval (CI) 1.4–8.4] or ever doing farm work (crude OR = 2.4; 95% CI, 1.4–3.6) were more likely to report diabetes. Ever working as a pesticide applicator was not associated with self-reported diabetes.

Statistically significant associations with diabetes were found for those with serum levels above the minimum detectable concentration for *trans*-nonachlor, oxychlordane, and β-HCH (Table 3). A statistically significant association is also evident among those with the highest serum concentration of *p,p*′-DDT (comparing those above the median level to those below the detectable limit) and *p,p*′-DDE (comparing those in the highest quartile to those in the lowest quartile of serum concentration). A dose–response relationship was evident for *trans-*nonachlor, oxy-chlordane, *p,p*′-DDT and *p,p*′-DDE.

Stratified analysis provided some evidence of possible interactions. There was evidence of effect modification by BMI for those with the highest quartile of serum concentration of *p,p*′-DDE (obese OR = 5.0; 95% CI, 0.8–30.3; nonobese OR = 1.8; 95% CI, 0.6–5.2), and for those with above the median level of β-HCH serum concentration (obese OR = 0.9; 95% CI, 0.1–6.6; nonobese OR = 4.6; 95% CI, 1.4–15.0). There was evidence of effect modification by sex for dieldrin (male OR = 0.6; 95% CI, 0.1–2.9; female OR = 4.1; 95% CI, 1.1–14.5), and smoking status for HCB (current smoker OR = 1.1; 95% CI, 0.4–3.2; former smoker OR = 0.4; 95% CI, 0.1–2.7; never smoker OR = 3.0; 95% CI, 1.1–8.1).

Because of missing data for serum total cholesterol or serum triglycerides, 11.3% of the study sample is missing data for total lipids. Persons with self-reported diabetes and those with pesticide levels above the detectable limit were significantly more likely to have missing values. Analysis of the association between self-reported diabetes and pesticide concentrations corrected for total lipids was performed with a reduced study sample size (*n* = 1,132). The reduction in sample size created cell sizes too small for stable parameter estimation for some organochlorine pesticides (Table 4). The relationship between *p,p*′-DDT and self-reported diabetes remained statistically significant among those with the highest level of exposure (OR = 2.3; 95% CI, 1.1–5.0, trend *p*-value = 0.04). Mean log-transformed serum glucose levels were significantly higher among those with measurable exposure levels of *trans*-nonachlor and the highest exposure levels of β-HCH compared with those below the detectable limit (Table 5).

## Discussion

The use of the HHANES database allowed an evaluation of the association between various organochlorine compounds and diabetes at a time when the use of organochlorine pesticides was recently discontinued in the United States and still being phased out in Mexico. A higher proportion of the HHANES sample had detectable levels of organochlorine pesticides when compared with national estimates of pesticide serum levels among the general United States population during a similar time period (1976–1980) from the second National Health and Nutrition Examination Survey (NHANES) (Stehr-Green 1989). Forty percent of the HHANES sample population was born outside the United States; lack of enforceable rules and regulations to control the use of pesticides in developing countries may have caused extensive environmental exposure (Albert et al. 1980). Comparisons to more recent biomonitoring results from NHANES 1999–2002 demonstrate the dramatic decline in measurable levels of organochlorine pesticides in the both the Mexican-American and the general populations (CDC 2005).

The results of this study need to be interpreted with caution because of the lack of specificity of the association between various organochlorine compounds and diabetes. The most studied organochlorine compound in its relation to diabetes is TCDD; dioxin was not measured in the HHANES population and the proportion of subjects with detectable levels of PCBs was < 1%. Therefore, we did not evaluate these compounds for their association with diabetes in our study. Our results were analogous to those of studies using more recent health survey data sets performed in the United States which also found an association between diabetes and various organochlorine compounds, including *p,p*′-DDT, *p,p*′-DDE, oxychlordane, and *trans*-nonachlor (Everett et al. 2006; Lee et al. 2006). Because of considerable correlation between serum concentrations of organochlorine pesticides, drawing conclusions about associations with individual compounds is difficult. The association between serum organochlorine concentrations and self-reported diabetes may be stronger for those exposed to more than one organo-chlorine pesticide (Lee et al. 2006).

When corrected for serum lipids, only the association between self-reported diabetes and serum concentration of *p,p*′-DDT remained statistically significant. Missing values for total lipids were more common among those with higher serum levels and persons with self-reported diabetes; this would most likely have created a selection bias towards the null.

Body weight may play an important yet undetermined role in the association between organochlorine compounds and diabetes. The direction of the association of BMI with serum levels of organochlorines is not clear; both positive and negative associations have been reported in the literature (Glynn et al. 2003; James et al. 2002; Laden et al. 1999; Moysich et al. 2002; Schildkraut et al. 1999; Wolff et al. 2005). Serum levels of organochlorine compounds may be affected by recent weight loss due to lipid mobilization (Pelletier et al. 2003). In our study, the association between diabetes and *p,p*′-DDE appeared stronger among those with high BMI, whereas the association between diabetes and β-HCH was stronger among those who were not obese. Previous studies have also found evidence of effect modification by body weight (Lee et al. 2006); however, those results as well as ours are limited by a lack of data on recent weight loss.

Family history of diabetes was not available in this data set, but may have served as a possible effect modifier. A genetic epidemiology study conducted among banana farmers in Costa Rica found that farmers who inherited “unfavorable” metabolizing genes had significantly more adverse biological effects than controls or farmers who had inherited “favorable” alleles (Au et al. 1999). Molecular indicators of the diabetogenic effects of dioxin exposure were more pronounced among those with known diabetes risk factors such as obesity and family history of disease (Fujiyoshi et al. 2006). Genetic and physiologic risk factors for diabetes may factor in the mechanism by which organochlorine exposure is associated with diabetes.

Serum concentrations of organochlorine pesticides, including oxychlordane and *trans*-nonachlor, have been associated with increased insulin resistance (Lee et al. 2007). Impaired glucose metabolism has been proposed as a possible mechanism for the association between diabetes and organochlorine pesticide exposure; however experimental investigations have focused on dioxin-like compounds and organophosphate pesticides (Abdollahi et al. 2004; Enan and Matsumura 1994; Enan et al. 1996; Remillard and Bunce 2002). In the design of HHANES, a subsample of persons 20–74 years of age were assigned to the oral glucose tolerance test, and the remainder were assigned to the pesticide subsample. Therefore, the glucose values used were nonfasting levels and can be used only as a crude proxy for glucose abnormalities. Insulin levels were not available.

The major limitation of this study is the cross-sectional design. The association between lipid soluble toxins and diseases like diabetes that have lipid abnormalities may be partly explainable by reverse causality. Diabetes may result in a greater solubility of organochlorines per unit total serum lipid and cause an association with organochlorine pesticides (Longnecker and Michalek 2000). The metabolic clearance of some drugs is altered by diabetes (Gwilt et al. 1991). It is possible that diabetes alters the pharmokinetics of pesticide elimination resulting in higher pesticide levels among diabetics. However, a study of TCDD elimination rates among U.S. Air Force veterans did not detect any difference by diabetes status (Michalek et al. 2003).

In an effort to partially address the issue of temporality, we examined the association between occupations presumed to involve pesticide exposure and prevalence of diabetes. Thus, actual pesticide levels (which may be altered by diabetes) are not used. Rather, occupation, which is unlikely to be a consequence of diabetes, is used as a proxy for pesticide exposure. Working in a pesticide plant and farming occupation were each associated with a higher prevalence of diabetes. These occupations were also associated with higher levels of β-HCH, HCB, *p,p*′-DDT, and oxychlordane.

In previous studies, organochlorines were associated with type 2 and type 1 diabetes. Small sample size prevented stratification by type of diabetes, but 90–95% of cases are attributed to type 2 diabetes in the United States (Longnecker and Daniels 2001). The use of self-reported diabetes and the inability to link pesticide exposure data to oral glucose tolerance test results resulted in an underestimation of diabetes in this population. Previous work using HHANES has shown that the prevalence of undiagnosed diabetes is 1.8% among Mexican Americans 20–44 years of age and 9.6% among those 45–74 (Perez-Stable et al. 1989). If the underreporting is more pronounced among the more highly exposed (jobs of lower socioeconomic status are generally more likely to have higher exposures), then the magnitude of the association between diabetes and serum levels of organochlorine pesticides would be underestimated in this population.

In conclusion, this study contributes to the growing literature examining the association between organochlorine pesticides and diabetes mellitus. A positive association that increased with serum concentration was found for most of the compounds evaluated. Future studies should focus on clarifying the temporal sequence of the association and examining the role of serum lipids, recent weight loss, obesity, and family history of diabetes.

## Figures and Tables

**Table 1:** Whole weight organochlorine serum concentrations, Mexican Americans 20–74 years of age, HHANES 1982–1984 (*n* = 1,303).

Organochlorine pesticide	Minimum detection limit (ppb)	No. (%) above detection limit	Minimum–maximum (ppb)^a^	Median (ppb)^a^	Mean (ppb)^a^
Hexachlorobenzene	1.00	78 (5.7)	1.00–3.92	1.34	1.63
Transnonachlor	1.00	146 (9.7)	1.00–17.60	1.52	2.16
*p*,*p*′-DDT	2.00	272 (18.1)	2.00–71.92	3.22	4.78
*p*,*p*′-DDE	1.00	1,300 (99.8)	1.98–228.22	9.00	28.09
β-HCH	1.00	344 (22.5)	1.00–13.68	1.70	2.14
Oxychlordane	1.00	82 (5.5)	1.00–6.60	1.22	1.69
Dieldrin	1.00	67 (4.8)	1.00–9.00	1.50	1.79

a Includes only values above detection limit.

**Table 2:** Demographics of Mexican Americans 20–74 years of age by self-reported diabetes, HHANES, 1982–1984 (*n* = 1,303).^a^

Characteristic	Sample size (*n* = 1,303)	Self-reported diabetes (*n* = 89) [no. (%)]	No self-reported diabetes (*n* = 1,214) [no. (%)]	*p*-Value (χ^2^)
BMI				0.0076
< 25.0 (normal or underweight)	531	14 (2.0)	517 (98.0)	
25.0–29.9 (overweight)	504	40 (6.0)	464 (94.0)	
> 29.9 (obese)	268	35 (12.1)	233 (87.9)	
Age (years)^b^				0.0014
20–44	831	15 (1.6)	816 (98.4)	
45–54	256	21 (7.2)	235 (92.8)	
55–64	137	33 (25.6)	104 (74.4)	
65–74	79	20 (25.6)	59 (74.5)	
Sex				0.14
Female	775	55 (6.3)	720 (93.7)	
Male	528	34 (4.6)	494 (95.4)	
Place of birth				0.38
U.S. born	756	57 (6.2)	699 (93.8)	
Foreign born	547	32 (4.3)	515 (95.7)	
Education				0.024
< High school	560	63 (10.4)	497 (89.6)	
High school	507	20 (2.6)	487 (97.4)	
> High school	217	6 (2.4)	211 (97.6)	
Smoker				0.099
Current	400	26 (5.0)	374 (95.0)	
Former smoker	257	26 (8.3)	231 (91.7)	
Never smoker	643	37 (4.6)	606 (95.4)	
Alcohol				0.0050
Nondrinker	584	54 (8.4)	530 (91.6)	
Former drinker	62	11 (16.4)	51 (83.6)	
Current	655	24 (2.6)	631 (97.4)	
Poverty index (percentile)				0.030
< 25th (< 0.99)	433	38 (7.4)	395 (92.6)	
25–75th (0.99–2.74)	601	42 (5.5)	559 (94.5)	
> 75th (> 2.74)	269	9 (2.7)	260 (97.3)	

a Frequencies are weighted to the Mexican-American population of the U.S. Southwest.

b Age at time of examination.

**Table 3:** Association of self-reported diabetes with whole weight serum organochlorine concentrations, HHANES, 1982–1984 (*n* = 1,303).

Pesticide	No.	No. self-reported diabetes	Crude OR (95% CI)	Age-adjusted OR (95% CI)	Confounder-adjusted OR (95% CI)
Hexachlorobenzene					
≥ 1.00 ppb (> MDL)	78	9	1.6 (1.2–2.3)	1.3 (0.7–2.4)	1.5 (0.9–2.4)^a^
< 1.00 ppb (< MDL)	1,225	80	1.0	1.0	1.0
Dieldrin					
≥ 1.00 ppb (> MDL)	67	15	5.8 (1.8–18.5)	3.3 (1.1–10.2)	2.2 (0.8–6.6)^b^
< 1.00 ppb (< MDL)	1,236	74	1.0	1.0	1.0
*trans*-Nonachlor
≥ 1.00 ppb (> MDL)	146	30	6.1 (3.2–11.9)	2.6 (1.2–5.5)	2.9 (1.3–6.4)^a^
< 1.00 ppb (< MDL)	1,157	59	1.0	1.0	1.0
> 1.80 ppb (> median^c^)	55	13	7.1 (3.2–15.6)	2.9 (1.0–8.2)	3.4 (1.4–7.9)
1.00–1.80 ppb (< median)	91	17	5.6 (2.4–2.6)	2.4 (1.0–5.7)	2.6 (0.9–7.2)
< 1.00 ppb (< MDL)	1,157	59	1.0	1.0	1.0
Oxychlordane					
≥ 1.00 ppb (> MDL)	88	22	6.7 (2.9–15.8)	2.7 (0.9–7.9)	3.1 (1.1–9.1)^a^
< 1.00 ppb (< MDL)	1,221	67	1.0	1.0	1.0
> 1.265 ppb (> median^c^)	39	11	7.5 (3.6–15.8)	3.0 (1.2–7.5)	3.3 (1.2–8.7)
1.00–1.265 ppb (< median)	43	11	6.1 (2.1–17.9)	2.4 (0.6–10.1)	3.0 (0.7–12.5)
< 1.00 ppb (< MDL)	1,221	67	1.0	1.0	1.0
*p,p*′-DDT					
≥ 2.00 ppb (> MDL)	272	40	4.1 (2.0–8.4)	2.4 (1.2–5.1)	1.9 (1.0–3.7)^b^
< 2.00 ppb (< MDL)	1,031	49	1.0	1.0	1.0
> 3.70 ppb (> median^c^)	110	19	4.9 (2.3–10.6)	3.8 (1.5–9.7)	2.9 (1.2–6.8)
2.00–3.70 ppb (< median)	162	21	3.5 (1.4–9.0)	1.8 (0.7–9.7)	1.4 (0.5–4.0)
< 1.00 ppb (< MDL)	1,031	49	1.0	1.0	1.0
*p,p*′-DDE					
> 58.60 ppb (> 75th percentile^c^)	142	22	6.5 (2.9–14.7)	2.63 (1.2–5.8)	
39.10–58.60 ppb (50th–75th percentile)	173	22	5.5 (2.4–12.6)	2.43 (1.0–5.7)	
22.81–39.10 ppb (25th–50th percentile)	314	22	2.6 (0.9–7.6)	1.42 (0.6–3.7)	
< 22.81 ppb (< 25th percentile)	671	23	1.0	1.0	
β-HCH					
≥ 1.00 ppb (> MDL)	344	53	6.7 (2.9–15.8)	2.3 (1.1–5.0)	2.1 (1.0–4.3)^a^
< 1.00 ppb (< MDL)	959	36	1.0	1.0	1.0
> 2.10 ppb (> median^c^)	126	25	7.5 (3.6–15.8)	2.9 (0.9–8.8)	2.7 (0.9–8.2)
1.00–2.10 ppb (< median)	218	28	6.1 (2.1–17.9)	2.0 (1.1–3.6)	1.7 (1.1–2.8)
< 1.00 ppb (< MDL)	959	36	1.0	1.0	1.0

MDL, minimum detectable limit.

a ORs adjusted for age at examination and BMI.

b ORs adjusted for age at examination, BMI, alcohol consumption.

c Based on distribution among self-reported diabetics.

**Table 4:** Association of self-reported diabetes with serum organochlorine concentrations, corrected for total lipids, HHANES, 1982–1984 (*n* = 1,132).

Pesticide	No.	No. self-reported diabetes	Crude OR (95% CI)	Age-adjusted OR (95% CI)	Confounder-adjusted OR (95% CI)
*trans*-Nonachlor					
> 179.22 ppb (> median^a^)	65	9	3.6 (1.0–12.9)	1.2 (0.3–4.4)	1.5 (0.4–4.9)^b^
≤ 179.22 ppb (< median)	29	6	7.3 (1.6–34.6)	2.9 (0.5–15.8)	3.6 (0.5–25.6)
Below detectable limit	1,038	50	1.0	1.0	1.0
Oxychlordane					
> 143.75 ppb (> median^a^)	27	6	5.4 (1.1–26.1)	1.5 (0.3–8.5)	2.2 (0.4–14.3)^b^
≤ 143.75 ppb (< median)	14	4	10.9 (1.3–89.9)	3.7 (0.1–93.9)	3.6 (0.1–120.7)
Below detectable limit	1,091	55	1.0	1.0	1.0
*p,p*′-DDT					
> 496.36 ppb (> median^a^)	103	15	4.6 (2.1–10.0)	3.3 (1.5–7.3)	2.3 (1.1–5.0)^c^
≤ 496.36 ppb (< median)	107	13	3.9 (1.8–8.2)	1.9 (0.9–4.0)	1.5 (0.7–3.1)
Below detectable limit	922	37	1.0	1.0	1.0
*p,p*′-DDE					
> 6,749.90 ppb (> 75th percentile^a^)	180	19	3.3 (1.4–7.4)	1.5 (0.8–2.9)	
5,232.35–6,749.90 ppb (50th–75th percentile)	116	15	4.5 (1.3–15.8)	1.8 (0.7–4.6)	
2,394.92–5,232.35 ppb (25th–50th percentile)	421	15	1.2 (0.4–3.5)	0.8 (0.3–2.3)	
< 2394.92 ppb (< 25th percentile)	415	16	1.0	1.0	
β-HCH					
> 280.53 ppb (> median^a^)	110	16	5.5 (2.2–13.7)	1.8 (0.6–5.2)	1.7 (0.6–4.6)^b^
≤ 280.53 ppb (< median)	141	16	3.9 (2.3–6.8)	1.6 (0.7–3.9)	1.4 (0.6–3.2)
Below detectable limit	881	33	1.0	1.0	1.0

a Based on distribution among self-reported diabetics.

b ORs adjusted for age at examination and BMI.

c ORs adjusted for age at examination, BMI, alcohol consumption.

**Table 5:** Mean^a^ logarithm serum glucose^b^ by organochlorine pesticide concentration, HHANES, 1982–1984.

Pesticide	Mean ± SE	*p-*Value^c^
*trans*-Nonachlor		
> 1.80 ppb (> median^d^)	4.76 ± 0.08	0.033
1.00–1.80 ppb (< median)	4.70 ± 0.05	0.027
< 1.00 ppb (< MDL)	4.57 ± 0.01	
Oxychlordane		
> 1.265 ppb (> median^d^)	4.72 ± 0.11	0.22
1.00–1.265 ppb (< median)	4.65 ± 0.06	0.22
< 1.00 ppb (< MDL)	4.58 ± 0.01	
*p,p*′-DDT		
> 3.70 ppb (> median^d^)	4.65 ± 0.05	0.20
2.00–3.70 ppb (< median)	4.64 ± 0.04	0.051
< 1.00 ppb (< MDL)	4.57 ± 0.01	
*p,p*′-DDE		
> 58.60 ppb (> 75th percentile^d^)	4.64 ± 0.04	0.081
39.10–58.60 ppb (50th–75th percentile)	4.58 ± 0.02	0.73
22.81–39.10 ppb (25th–50th percentile)	4.59 ± 0.03	0.45
< 22.81 ppb (< 25th percentile)	4.57 ± 0.01	
β-HCH		
> 2.10 ppb (> median^d^)	4.72 ± 0.04	0.0029
1.00–2.10 ppb (< median)	4.59 ± 0.02	0.088
< 1.00 ppb (< MDL)	4.56 ± 0.01	

MDL, minimum detectable limit

a Least-squares means adjusted for age at examination and BMI.

b Nonfasting, random serum glucose in milligrams per deciliter.

c *p*-Value for *t*-test testing difference of means.

d Based on distribution among self-reported diabetics.
